# Design modifications of high-flexion TKA do not improve short term clinical and radiographic outcomes

**DOI:** 10.1186/1471-2474-15-433

**Published:** 2014-12-15

**Authors:** Jai Gon Seo, Young-Wan Moon, Moon Jong Chang, Byung Chul Jo, Yong Beom Park, Deuk Soo Lim, Byung Hoon Lee

**Affiliations:** Department of Orthopaedic Surgery, Samsung Medical Center, Sungkyunkwan University School of Medicine, 81 Irwon-ro, Gangnam-gu, Seoul 135-710 South Korea

**Keywords:** Total knee arthroplasty, High-flexion knee, Outcome scores, Range of motion

## Abstract

**Background:**

The prosthesis of contemporary total knee arthroplasty (TKA) has been modified to provide a more familiar environment for higher flexion angle of the replaced knee. The design modifications continue based on evidence reported in the literature. However, whether these modifications of the prosthesis design lead to improvements in clinical results needs further investigation. We determined whether the prosthesis modifications based on recent evidence improve clinical and radiographic results following high flexion TKA.

**Methods:**

524 patients who underwent primary TKA using two different high flexion prostheses were divided to Group 1 (HF-1) using a high flexion prosthesis, group 2 (HF-2) using the more recently devised high flexion prosthesis, which claims to be adopted from evidence proposed in the literature. Clinical outcomes included ranges of motion (ROM), the Knee Society knee and function score (KSKS and KSFS), the Western Ontario and McMaster Universities Arthritis Index (WOMAC) score, radiologic evaluation, and complication related to surgery.

**Results:**

No differences in terms of clinical and radiographic results were observed between the groups at the 2 year follow-up. The mean ROM was 123°and 124° in the HF-1 and HF-2 groups, respectively. KSKS were 90 and 89.1, KSFS were 76.6 and 81.8, and total WOMAC scores were 23.1 and 24.9 in the HF-1 and HF- 2 groups. No differences of the incidences of radiolucency on radiographs (1.4% in HF-1, 2.1% in HF-2) and dislocation (1 case in HF-1 only) was observed.

**Conclusions:**

Even if recent modifications in the design of high flexion TKA prosthesis were based on evidence in the literature, they did not provide meaningful improvements in short-term clinical and radiographic outcomes after TKA. Surgeons should consider our findings when choosing a prosthesis for their patients.

**Electronic supplementary material:**

The online version of this article (doi:10.1186/1471-2474-15-433) contains supplementary material, which is available to authorized users.

## Background

Total knee arthroplasty (TKA) is an effective method to eliminate pain and restore function in a patient with chronic arthritis of the knee joint. Despite excellent surgical outcomes and longevity of contemporary TKA, deep flexion of the knee after TKA may be still requested by patients, particularly Asians, who are accustomed to squatting and sitting on the floor[1–3]. Many investigators suggest a multidisciplinary approach such as improving intraoperative technique and postoperative rehabilitation to achieve a greater range of motion (ROM) after surgery. Furthermore, prosthetic design changes have recently been introduced in an effort to gain higher flexion angles.

High flexion prostheses incorporate several common kinematic modifications compared to traditional designs to improve kinematics at higher flexion angles[4–6]. These devices have an extended sagittal curve and a 2– 3 mm thicker posterior femoral condyle to maintain contact area and reduce stress on the insert at higher flexion angles[7]. The tibial post is located 1–2 mm more posteriorly to guide femoral rollback during high flexion. Furthermore, the cam is extended to the surface of the femoral component posteriorly to increase the articular contact area at higher flexion angles[8]. The anterior face of the polyethylene tibial bearing has also been cut out to reduce patellar tendon impingement during high degrees of flexion.

However, it has been controversial whether the aforementioned theoretical improvements in design result in clinical improvements. The advantages have been demonstrated in some *in vivo* analyses, and several authors have reported improved postoperative ROM compared with that of the conventional designs[2, 9–12]. In contrast, other studies have revealed a high rate of aseptic loosening of the femoral component during high flexion TKA and an increased rate of dislocation during a high-flexion angle at the short-term follow up. Thus, more attention was paid to the cam-post engagement design and the amount of posterior condyle resection after reports of high incidence of early loosening and dislocation[10, 13, 14]. Thus, implant manufacturers have been striving to assure implant safety and provide improved designs according to evidence reported in the literature. However, it is still controversial whether the modified implants in high flexion designed knee prostheses can actually affect clinical results.

We determined whether these theoretical improvements in implant design improved postoperative ROM, clinical outcome, and reduced complications such as osteolysis and dislocation following contemporary high flexion TKA. We hypothesized that the design modifications would affect postoperative clinical outcomes and complications after TKA.

## Methods

We retrospectively investigated 647 patients who underwent primary TKA with two different high flexion prostheses from January 2011 to April 2012 at our institution. All patients were followed up for more than 2 years after surgery.

Two high-flexion designed total knee prostheses (LOSPA, Corentec, Inc. South Korea; Scorpio Non-Restrictive Geometry (NRG), Stryker, NJ, USA) were used. Two prostheses were used bimonthly.

**Figure 1:**
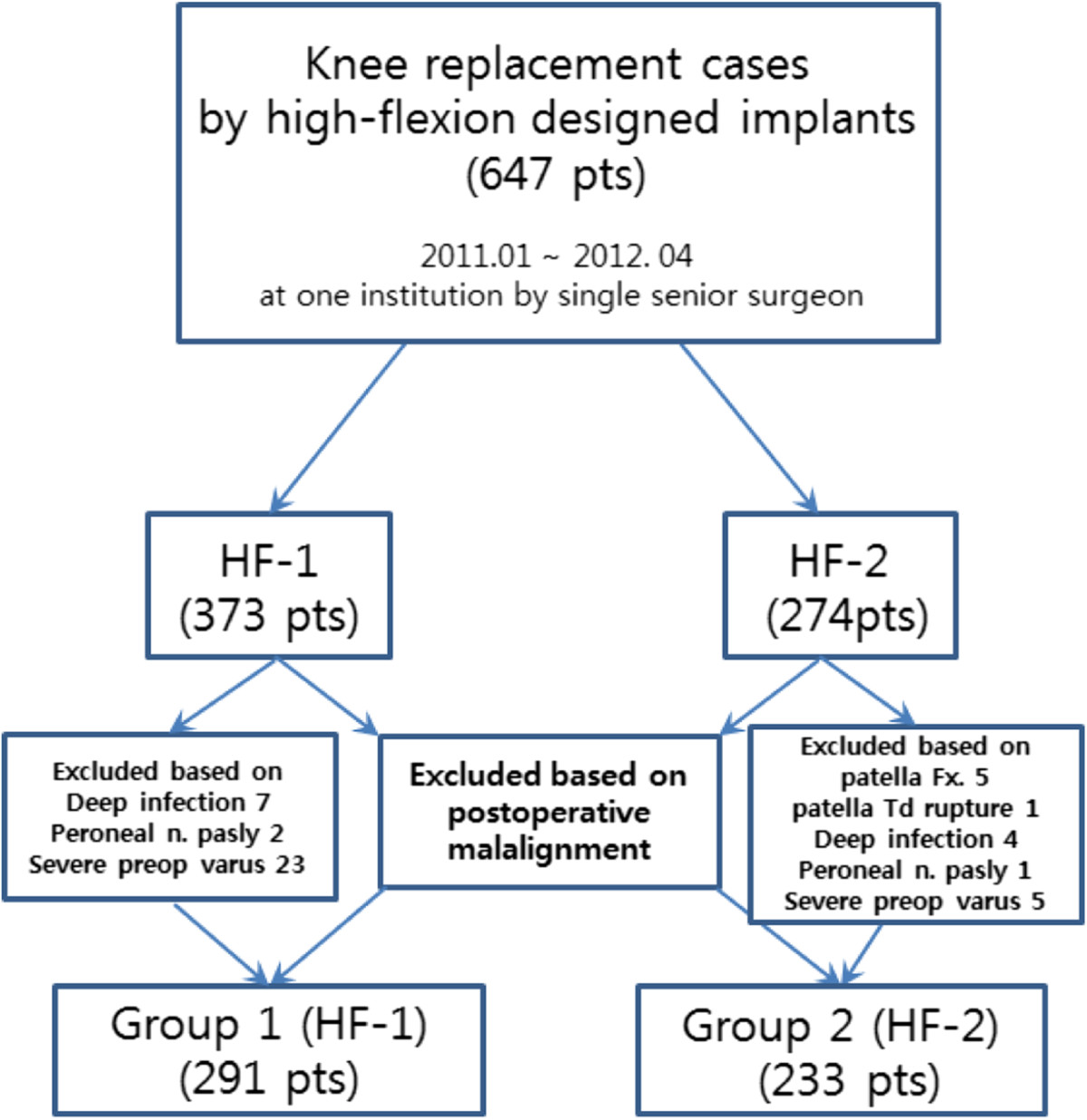
**A total of 647 patients were initially eligible for inclusion, and 524 patients were included; the schematic shows subject involvement in the study.**

The patients were divided into two groups according to the implant type used. Group 1 (HF-1, Scorpio NRG) consisted of 373 patients who underwent TKA using a high flexion implant, and group 2 (HF-2, LOSPA) was comprised of 274 patients who received a modified prosthesis, which was devised more recently, based on evidence from the literature. Before analysis, we included only those patients who were between 3° of valgus and varus in terms of the mechanical femoro-tibial angle (MFTA) after implantation, which is one of the factors affecting postoperative ROM, early loosening, and outcome[15, 16]. Thus, 323 patients in HF-1 (MFTA: mean 1.2°, standard deviation 1.4°) and 249 patients in HF-2 (MFTA: mean 1.5° and standard deviation 2.3°) were registered in this investigation. No significant differences were observed between the groups with regard to the position of the femoral and tibial components in the coronal and sagittal planes or coronal limb alignment on preoperative radiographs (data not shown). Additionally, patients who had postoperative complications that may have had a negative impact on clinical outcome such as patellar fracture or periprosthetic infection (nine patients in the HF-1 group and 11 patients in the HF-2 group) and patients with complex knees and preoperative ROM < 50°, severe varus or valgus deformity > 20° combined with a bone defect requiring bone grafting were excluded. Consequently, 524 patients (291 in the HF-1 and 233 in the HF-2) were included (Figure 1). No demographic differences were observed between the groups (Table 1). The current study obtained Institutional Review Board approval from our institution (Samsung Medical Center, 2013-06-098) and written informed consent was obtained from all participants.

**Table 1:** **Comparison of preoperative demographics and clinical status between the groups**

Parameters	HF-1 group (N = 291)	HF-2 group (N = 233)	***P*** value
Sex (proportion of female patients)	263 (90%)	213 (91%)	NS (0.141)
Age (year)	68 ± 6.4	69 ± 6.6	NS (0.125)
Preoperative MFTA (°)	9.4 ± 5.4	9.5 ± 4.8	NS (0.816)
Preoperative total WOMAC score	55.9 ± 17.8	55.6 ± 16.5	NS (0.725)

NS: not significant, ROM: range of motion, MFTA: mechanical femoro-tibial angle, WOMAC: Western Ontario and McMaster Universities Index

Values are mean ± standard deviation (*P* < 0.05).

All operations were performed by a single senior surgeon (one of the authors), and all TKAs were performed using an extramedullary femoral and tibial guide system[17]. All the components were cemented with Simplex P (Howmedica, Rutherford, New Jersey) bone cement, and all the patellae were resurfaced with an all polyethylene dome-shaped component, implanted with bone cement. Quadriceps-strengthening exercises were started immediately after surgery as basic postoperative rehabilitation, and patients began walking with use of a walker on the first postoperative day. The second postoperative day, they started active and passive range-of-motion exercises under the supervision of a physical therapist. Weight bearing high-flexion activities such as squatting were allowed as tolerated.

All clinical and radiographic evaluations were performed by an independent investigator at each follow up visit, which were scheduled at 2 months, 1 year, and annually thereafter. At 1 year follow-up, the maximum flexion range of knee movement was measured by a physician assistant who was blinded to the study design, using a standard goniometer with the patient in the supine position on a table. The Knee Society Knee and Function score (KSKS and KSFS)[13] and the Western Ontario and McMaster Universities Arthritis Index (WOMAC) index score were obtained[18, 19]. Surgical complications that occurred within the follow-up period were also recorded. At 2 year follow-up, the incidence of radiological change such as progressive radiolucency was analyzed for evaluation of early loosening after TKA.

Standing anteroposterior (AP), lateral, and Merchant’s view radiographs were obtained at every follow up, and mechanical femorotibial alignment was measured on full limb standing AP radiographs using a picture archiving and communication system (General Electric, Milwaukee, WI, USA). All radiographs were made with standard positioning (directing the patella anteriorly and with a focal film distance of 100 cm), which were analyzed using the Knee Society radiological scoring system to delineate radiolucency around the component[19]. A radiolucent line > 1 mm on the bony contact resurface zone of the femoral component at the 2 year follow up was considered radiolucency to determine how the implant design’s modifications affected radiographic results. We compared the incidences between the two groups.

### Theoretical differences in design between the two high flexion implants

**Figure 2:**
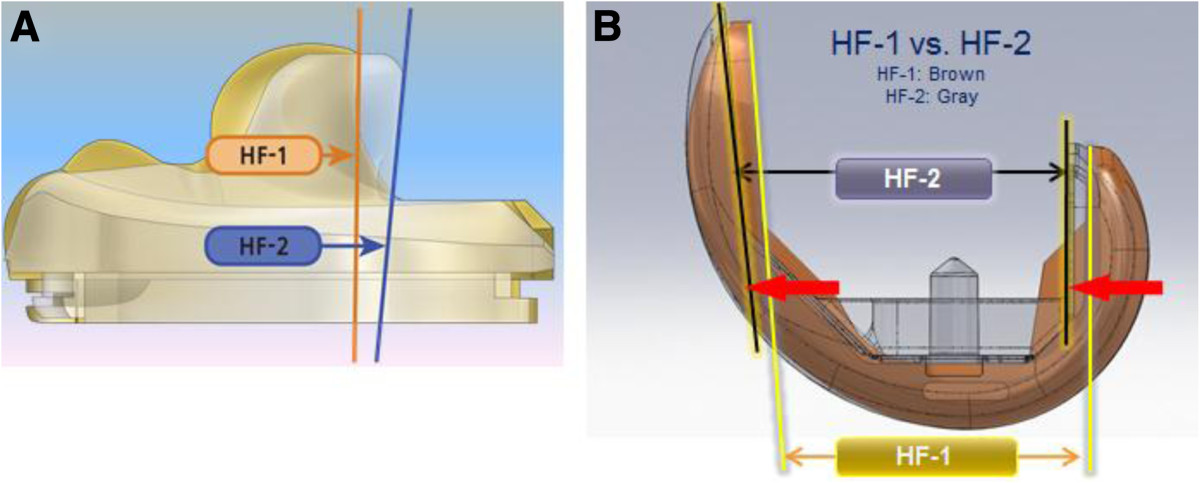
**The HF-2 was modified to provide safe and adequate flexion in contrast to the HF-1 device in the cam-post mechanism (A) and femoral component design (B).**

The two designs of implant incorporated modifications to the geometry of the design intended to improve postoperative ROM and provided safe and adequate flexion by preventing loading on the edge of the posterior tibial articular surface and by increasing the tibiofemoral contact area during high flexion[8, 20]. They have common characteristics in their design. That is, the femoral component of the posterior stabilized HF-1 and HF-2 knee prostheses had a single AP femoral radius, a deepened patella-femoral groove, which provided secure guidance of the patella, increased flexion, and reduced peak stress throughout ROM. In contrast, the HF-2 design had some additional modifications in the femoral component. While the posterior radius in the HF-1 femoral component was 8 mm, it was 10 mm in the HF-2 device, leading to increased contact area and higher posterior support length. In contrast, the anterior flanged angle was designed higher by 5° in the HF-2 compare to 3° in the HF-1 device, which was intended to reduce the amount of anterior bone resection. In other words, the HF-2 design preserved more bone anteriorly, and resulted in the same amount of bone loss and a larger posterior radius than those of the HF-1 implant to decrease contact stress (Figure 2). This may reduce loads in the knee during deep flexion and result in less wear or loosening on the insert. Last, both implants were designed to heighten jump distance and prevent exceeding the cam post by rollback at deep flexion. However, the HF-2 design was modified to be extended proximally and moved to the posterior direction to create an inverse slope on the tibial posterior and posterior released articular surface for safety during deep flexion.

The statistical analysis was performed using SAS ver. 9.13 (SAS Institute, Cary, NC, USA). The ROM, clinical outcomes (KSKS, KSFS, and WOMAC subscale scores) and radiographic MFTA of patients are described as means and standard deviations. Differences were compared between the two groups by Student’s *t*-test. The incidence of osteolysis was numbered and compared to the statistical significance determined by Pearson’s chi-square analysis or Fisher’s exact test. The threshold for significance was < 0.05. The statistical analysis in this study had > 80% power to detect a 10° difference in postoperative ROM between the groups (accepting < 5% probability of a type I error). The authors set the score difference according to a previous study[21].

## Results

The HF-2 group did not show greater postoperative flexion of the knee and improved knee scores than that of the HF-1 group. The mean preoperative angle of flexion of the knee was 123° in the HF-1 group and 124° in the HF-2 group, and the mean postoperative angle of flexion improved to 129° and 127°, respectively (*P* = 0.098) (Table 2). No differences in the postoperative KSKS or total WOMAC scores were observed between the two groups at the 1 year follow-up (*P* = 0.448 and *P* = 0.093, respectively). The mean postoperative KSKS were 90.0 in the HF-1 and 89.1 in the HF-2 group, and total WOMAC scores were 23.1 and 24.9 in the HF-1 and HF-2 groups, respectively (Table 3). However, significant differences were observed in the KSFS and stiffness on the WOMAC subscales, The HF-2 group showed more improved results than those of the HF-1 on the KSFS (HF-1, 76.6 vs. HF-2, 81.8, *P* < 0.001), whereas worse results were observed on the WOMAC stiffness subscale (HF-1, 2.3 vs. HF-2, 2.7, *P* = 0.025).

**Table 2:** **Mean range of motion at preoperative and postoperative 1 year**

Parameters	HF-1 group (N = 291)	HF-2 group (N = 233)	***P*** value
Preoperative ROM (^o^)	122.9 ± 16.6	123.7 ± 17.3	NS (0.512)
Postoperative ROM at 1 Year (^o^)	128.9 ± 10.3	126.9 ± 10.3	NS (0.098)

SD standard deviation, NS: no significant.

Values are mean ± standard deviation.

**Table 3:** **Postoperative clinical outcomes at the 1 year follow-up**

Parameters	Follow up at one year (Mean ± SD)		
	HF-1 group (N = 291)	HF-2 group (N = 233)	***P*** value
KSKS	90.0 ± 10.7	89.1 ± 8.8	NS (0.448)
KSFS	76.6 ± 13.5	81.8 ± 12.7	<0.001
WOMAC	23.1 ± 12.8	24.9 ± 14.4	NS (0.093)
Pain	2.3 ± 3.0	2.5 ± 3.1	NS (0.307)
Function	18.6 ± 9.7	19.7 ± 10.7	NS (0.141)
Stiffness	2.3 ± 1.8	2.7 ± 2.0	0.025

SD standard deviation, NS: no significant, KSKS: Knee Society Score, KSFS: Knee Society function score, WOMAC: Western Ontario and McMaster Universities Index.

Postoperative data were checked at the outpatient department 1 year postoperatively.

No significant difference was observed in the postoperative complication rates such as radiographic changes of progressive radiolucency or dislocation at the short term follow-up. The incidence rate of the radiolucency radiographic abnormality of osteolysis in the femoral component did not differ between the two groups on AP and lateral radiographs at the 2 year follow-up (*P* = 0.570). Radiographic changes were observed in four knees in the HF-1 and five knees in the HF-2 group. All osteolytic changes in the bone contact resurface zone of the femoral component were involved in zone 4 area. We found no significant difference in dislocation occurrence between the groups. One revision operation due to a femoro-tibial dislocation was found in HF-1 group, but no dislocations were observed in the HF-2 group.

## Discussion

Many implant suppliers are considering biomechanical aspects in their implant designs to provide theoretical advantages of a high flex design and achieve clinical improvements. We hypothesized that the design modifications in the high flexion TKA devices would provide increased ROM and result in better clinical outcomes with fewer complications after TKA. Therefore, we conducted a retrospective comparative study to identify whether the modifications in implant design affected clinical and radiological follow-up results.

Many researchers have reported that high-flexion type implants result in improved postoperative ROM compared to that of a standard posterior substitution type prosthesis. One important difference was an additional bone cut from the posterior femoral condyle compared to the regular posterior substituted type design. The femoral component has an elongated and widened cam design to increase stability, maintain spine strength, facilitate rollback, and ultimately increase ROM. In fact, Bellemans et al. reported that the posterior condylar offset decreased by 2 mm, and that maximal obtainable flexion was reduced by a mean of 12.2°[22]. It was previously revealed that high-flex designed prostheses for TKA achieve increased flexion angles from 129.4 to 139°[7, 9–11]. However our study did not show different result in postoperative ROMs in both groups, which used single radius designed, but different such as posterior radius length, femoral component geometry. We did not fully explain the kinematic differences due to the different geometry of the components but we inferred that a kinematic pattern favoring posterior femoral rollback was not associated with a greater ROM, at least for the high-flexion prosthesis.

Furthermore, our findings did not support the hypothesis that modifying the implant design for the high-flex knee positively affects postoperative clinical outcomes. No significant differences were observed in the KSKS or WOMAC scores, but significant differences were found in the KSFS and the WOMAC subscale stiffness score. Although improved KSFS scores were obtained in the HF-2 group, it may be difficult to acknowledge clinically meaningful results. We put a construction on clinical outcome results in our study to three points. First, clinical outcomes after TKA are affected by several factors such as operative technique, postoperative care and rehabilitation except implanted component’s design[23–25]. Second, advances have reached in the aspect of intraoperative skill, prosthetic design, and postoperative care in contemporary TKA. Third, parameters used for assessing clinical outcome are probably too crude to reflect slight modifications. Thus, we did not demonstarte that design modifications of high-flex prosthesis would provides improved clinical outcomes.

The device used in the HF-2 group was designed based on several theoretical improvements for reducing the risk of early loosening. The important modifications in the femoral component design were to manage stress during deep flexion of the knee by using extended and augmented posterior condyles[26] and for maintaining bone support by increasing the anterior flange angle 3–5°. However, we found no difference in the incidence rate of radiographic changes such as progressive radiolucency in either groups. No cases of re-operation due to loosening occurred during the short-term follow-up in either group but differences in the incidence rate of radiolucency in zone 4 were observed three and five cases in HF-1 and 2 groups, respectively. Finally, based on retrospective data, we presumed that the change in polyethylene design to heighten jump distance might reduce the dislocation rate after surgery, but we could not detect a correlation between the implant modification, jump distance, and outcomes (Table 4). Arnout et al.[27] reported that a low jump distance can be associated with dislocation in a posterior stabilized knee prosthesis, and low jump distance is comprised of the relative position of the cam, post height, and a rounded post design. However, we suggest that the modification of the cam and post design be reconsidered as a higher jump distance leads to increased susceptibility to dislocation during knee flexion.

**Table 4:** **Incidence of complications such as radiographic change of osteolysis in the femoral component and dislocation at the short-term follow-up (2 yrs)**

Parameters (No.)	HF-1 group (N = 291)	HF-2 group (N = 233)	***P*** value
Incidence of progressive radiolucency (%)	4 (1.4%)	5 (2.1%)	NS (0.570)
Dislocation	1	0	NA

NS: not significant, NA: not applicable.

Our study had some inherent limitations because of its retrospective design. The rather short follow up period of 2 year was also a limitation to judge early loosening. The parameters for assessing clinical outcome may be too crude to reflect the slight modifications, and we had a female dominant cohort. Nevertheless, we tried to overcome these limitations by comparing a relative uniform high-volume, matched by tight criteria for classifying radiological change such as progressive radiolucency in zone 4[28]. Accordingly, a prospective, randomized study is required to determine whether the implant design modification’s affect outcomes.

## Conclusions

Recent modifications in the design of high flexion TKA prostheses are based on evidence in the literature, but we were unable to detect meaningful improvements in short-term clinical and radiographic outcomes after TKA. Surgeons should consider our findings when choosing a prosthesis for their patients.

## Authors’ original submitted files for images

Below are the links to the authors’ original submitted files for images. Authors’ original file for figure 1 Authors’ original file for figure 2

## Abbreviations

AP: Anteroposterior
HF: High-Flexion
KSKS: Knee Society Knee Score
KSFS: Knee Society Function Score
ROM: Range of motion
TKA: Total knee arthroplasty
WOMAC: Western Ontario and McMaster University Osteoarthritis Index.

## References

[1] Kim JM, Moon MS (1995). Squatting following total knee arthroplasty. Clin Orthop Relat Res.

[2] McCalden RW, MacDonald SJ, Bourne RB, Marr JT (2009). A randomized controlled trial comparing “high-flex” vs “standard” posterior cruciate substituting polyethylene tibial inserts in total knee arthroplasty. J Arthroplasty.

[3] Mulholland SJ, Wyss UP (2001). Activities of daily living in non-Western cultures: range of motion requirements for hip and knee joint implants. Int J Rehabil Res.

[4] Jones RE (2006). High-flexion rotating-platform knees: rationale, design, and patient selection. Orthopedics.

[5] Gupta SK, Ranawat AS, Shah V, Zikria BA, Zikria JF, Ranawat CS (2006). The P.F.C. sigma RP-F TKA designed for improved performance: a matched-pair study. Orthopedics.

[6] Barink M, De Waal MM, Celada P, Vena P, Van Kampen A, Verdonschot N (2008). A mechanical comparison of high-flexion and conventional total knee arthroplasty. Proc Inst Mech Eng H.

[7] Kim YH, Sohn KS, Kim JS (2005). Range of motion of standard and high-flexion posterior stabilized total knee prostheses: a prospective, randomized study. J Bone Joint Surg Am.

[8] Argenson JN, Scuderi GR, Komistek RD, Scott WN, Kelly MA, Aubaniac JM (2005). In vivo kinematic evaluation and design considerations related to high flexion in total knee arthroplasty. J Biomech.

[9] Bin SI, Nam TS (2007). Early results of high-flex total knee arthroplasty: comparison study at 1 year after surgery. Knee Surg Sports Traumatol Arthrosc.

[10] Huang HT, Su JY, Wang GJ (2005). The early results of high-flex total knee arthroplasty: a minimum of 2 years of follow-up. J Arthroplasty.

[11] Laskin RS (2007). The effect of a high-flex implant on postoperative flexion after primary total knee arthroplasty. Orthopedics.

[12] Sharma A, Komistek RD, Scuderi GR, Cates HE (2007). High-flexion TKA designs: what are their in vivo contact mechanics?. Clin Orthop Relat Res.

[13] Insall JN, Dorr LD, Scott RD, Scott WN (1989). Rationale of the Knee Society clinical rating system. Clin Orthop Relat Res.

[14] Jung WH, Jeong JH, Ha YC, Lee YK, Koo KH (2012). High early failure rate of the Columbus posterior stabilized high-flexion knee prosthesis. Clin Orthop Relat Res.

[15] Bonner TJ, Eardley WG, Patterson P, Gregg PJ (2011). The effect of post-operative mechanical axis alignment on the survival of primary total knee replacements after a follow-up of 15 years. J Bone Joint Surg (Br).

[16] Ritter MA, Berend ME, Harty LD, Davis KE, Meding JB, Keating EM (2004). Predicting range of motion after revision total knee arthroplasty: clustering and log-linear regression analyses. J Arthroplasty.

[17] Seo JG, Moon YW, Lim JS, Park SJ, Kim SM (2012). Mechanical axis-derived femoral component rotation in extramedullary total knee arthroplasty: a comparison between femoral transverse axis and transepicondylar axis. Knee Surg Sports Traumatol Arthrosc.

[18] Bellamy N, Buchanan WW, Goldsmith CH, Campbell J, Stitt LW (1988). Validation study of WOMAC: a health status instrument for measuring clinically important patient relevant outcomes to antirheumatic drug therapy in patients with osteoarthritis of the hip or knee. J Rheumatol.

[19] Gogia PP, Braatz JH, Rose SJ, Norton BJ (1987). Reliability and validity of goniometric measurements at the knee. Phys Ther.

[20] Sultan PG, Most E, Schule S, Li G, Rubash HE (2003). Optimizing flexion after total knee arthroplasty: advances in prosthetic design. Clin Orthop Relat Res.

[21] Han HS, Kang SB, Yoon KS (2007). High incidence of loosening of the femoral component in legacy posterior stabilised-flex total knee replacement. J Bone Joint Surg (Br).

[22] Bellemans J, Banks S, Victor J, Vandenneucker H, Moemans A (2002). Fluoroscopic analysis of the kinematics of deep flexion in total knee arthroplasty. Influence of posterior condylar offset. J Bone Joint Surg (Br).

[23] Bourne RB, Chesworth BM, Davis AM, Mahomed NN, Charron KD (2010). Patient satisfaction after total knee arthroplasty: who is satisfied and who is not?. Clin Orthop Relat Res.

[24] Dahm DL, Barnes SA, Harrington JR, Sayeed SA, Berry DJ (2008). Patient-reported activity level after total knee arthroplasty. J Arthroplasty.

[25] Noble PC, Conditt MA, Cook KF, Mathis KB (2006). The John Insall Award: patient expectations affect satisfaction with total knee arthroplasty. Clin Orthop Relat Res.

[26] Delp SL, Kocmond JH, Stern SH (1995). Tradeoffs between motion and stability in posterior substituting knee arthroplasty design. J Biomech.

[27] Arnout N, Vandenneucker H, Bellemans J (2011). Posterior dislocation in total knee replacement: a price for deep flexion?. Knee Surg Sports Traumatol Arthrosc.

[28] Ryu J, Saito S, Yamamoto K, Sano S (1993). Factors influencing the postoperative range of motion in total knee arthroplasty. Bull Hosp Jt Dis.

[CR29] The pre-publication history for this paper can be accessed here:http://www.biomedcentral.com/1471-2474/15/433/prepub

