# Supplementary figures and images for: Design modifications of high-flexion TKA do not improve short term clinical and radiographic outcomes
Source: BMC Musculoskelet Disord. 2014 Dec 15;15:433. doi: 10.1186/1471-2474-15-433 (PMC4301904; doi:10.1186/1471-2474-15-433)

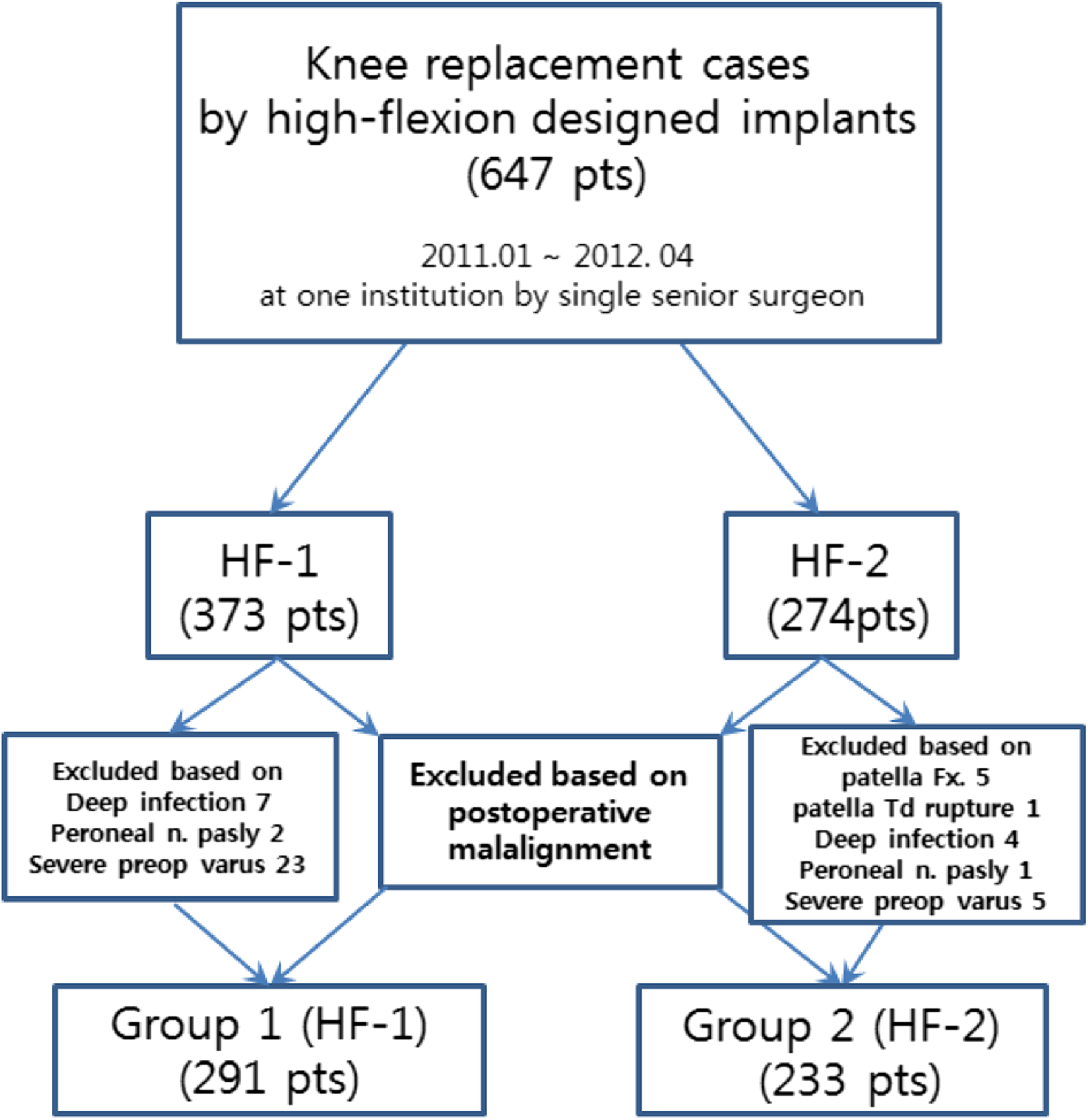

Supplement: Supplementary file 1 — Authors’ original file for figure 1 [file 12891_2014_2361_MOESM1_ESM.tif]

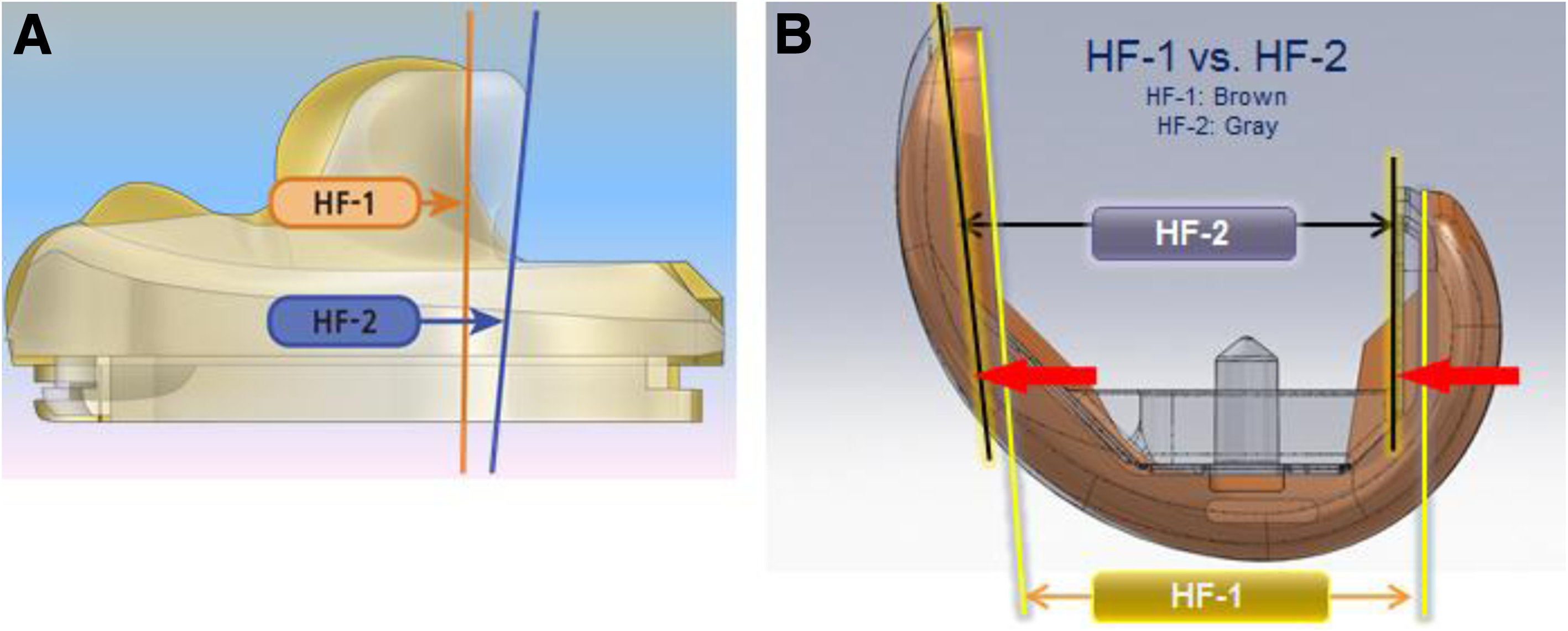

Supplement: Supplementary file 2 — Authors’ original file for figure 2 [file 12891_2014_2361_MOESM2_ESM.tif]
